# The genus *Weissella*: taxonomy, ecology and biotechnological potential

**DOI:** 10.3389/fmicb.2015.00155

**Published:** 2015-03-17

**Authors:** Vincenzina Fusco, Grazia M. Quero, Gyu-Sung Cho, Jan Kabisch, Diana Meske, Horst Neve, Wilhelm Bockelmann, Charles M. A. P. Franz

**Affiliations:** ^1^National Research Council of Italy, Institute of Sciences of Food ProductionBari, Italy; ^2^Department of Microbiology and Biotechnology, Max Rubner-InstitutKiel, Germany

**Keywords:** lactic acid bacteria, probiotic, prebiotic, bacteriocin, food safety, food quality, fermented food, detection and typing

## Abstract

Bacteria assigned to the genus *Weissella* are Gram-positive, catalase-negative, non-endospore forming cells with coccoid or rod-shaped morphology (Collins et al., 1993; Björkroth et al., 2009, 2014) and belong to the group of bacteria generally known as lactic acid bacteria. Phylogenetically, the *Weissella* belong to the *Firmicutes*, class *Bacilli*, order *Lactobacillales* and family *Leuconostocaceae* (Collins et al., 1993). They are obligately heterofermentative, producing CO_2_ from carbohydrate metabolism with either d(−)-, or a mixture of d(−)- and l(+)- lactic acid and acetic acid as major end products from sugar metabolism. To date, there are 19 validly described *Weissella* species known. *Weissella* spp. have been isolated from and occur in a wide range of habitats, e.g., on the skin and in the milk and feces of animals, from saliva, breast milk, feces and vagina of humans, from plants and vegetables, as well as from a variety of fermented foods such as European sourdoughs and Asian and African traditional fermented foods. Thus, apart from a perceived technical role of certain *Weissella* species involved in such traditional fermentations, specific *Weissella* strains are also receiving attention as potential probiotics, and strain development of particularly *W. cibaria* strains is receiving attention because of their high probiotic potential for controlling periodontal disease. Moreover, *W. confusa* and *W. cibaria* strains are known to produce copius amounts of novel, non-digestible oligosaccharides and extracellular polysaccharides, mainly dextran. These polymers are receiving increased attention for their potential application as prebiotics and for a wide range of industrial applications, predominantly for bakeries and for the production of cereal-based fermented functional beverages. On the detrimental side, strains of certain *Weissella* species, e.g., of *W. viridescens*, *W. cibaria* and *W. confusa*, are known as opportunistic pathogens involved in human infections while strains of *W. ceti* have been recently recongnized as etiological agent of “weissellosis,” which is a disease affecting farmed rainbow trouts. Bacteria belonging to this species thus are important both from a technological, as well as from a medical point of view, and both aspects should be taken into account in any envisaged biotechnological applications.

## A brief look at the history of *weissella* taxonomy

Collins and colleagues were the first to designate the genus *Weissella* in 1993 after taxonomic studies on atypical *Leuconostoc*-like microorganisms which stemmed from fermented sausages produced in Greece. Collins et al. (1993) noticed that these bacteria differed from other *Leuconostoc* species in a number of biochemical tests. Furthermore, molecular systematic investigations suggested that leuconostocs could be separated into three distinct genetic lineages, i.e., the genus *Leuconostoc sensu stricto*, the *L. paramesenteroides* group (which included also the atypical lactobacilli) and the species then known as *L. oenos* (which is currently classified as *Oenococcus oeni*). An in-depth study based on phenotypic, biochemical and 16S rRNA gene analyses allowed the differentiation of the new genus *Weissella* (gen. nov.) and the re-assignment of the species previously grouped in the genus *Lactobacillus* as *W. confusa*, *W. halotolerans*, *W. kandleri*, *W. minor*, and *W. viridescens*. In addition, one species previously assigned to the genus *Leuconostoc*, i.e., *W. paramesenteroides*, was also included in the new genus (Collins et al., 1993).

These species, as well as a newly described, coccus-shaped isolate *W. hellenica* reported in the study of Collins et al. (1993), all shared high 16S rRNA gene sequence similarity, warranting them to be included into the new genus *Weissella*. Unusual in this respect was that all *Lactobacillus* species at that time were considered to be of rod shape, while species of the genus *Leuconostoc* were often reported as cocci. Actually, the leuconostocs do not form perfectly round cells but are rather of lentil-like shape, i.e., with tapered ends, which Collins et al. (1993) referred to as “typical irregular coccoid morphology.” Nevertheless, the newly described genus *Weissella* comprises bacteria which are either cocci or rods in shape.

**Figure 1:**
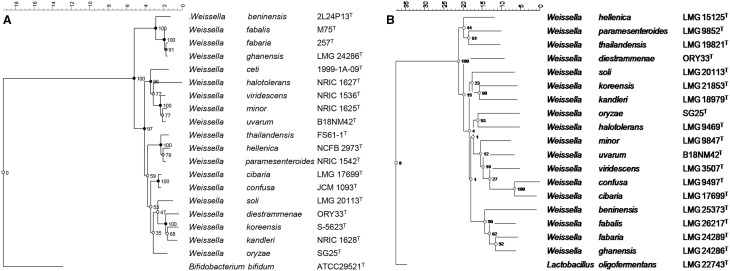
**Neighbor-joining phylogenetic tree based on (A) 16S rRNA sequences and (B) *phe*S gene sequences of *Weissella* species type strains**. The 16S rRNA sequence of *Bifidobacterium bifidum* was used as an outgroup sequence. Bootstrap values (%) derived from 1000 replicates are given at branch points. Bar indicates % sequence divergence.

Bacteria belonging to the genus *Weissella* are difficult to separate from members of the genera *Leuconostoc* or the heterofermentative lactobacilli on the basis of phenotypic characteristics only. As mentioned above, the taxonomy of the closely related bacteria in these groups, and the new description of the genus *Weissella*, was possible only on the basis of molecular taxonomical techniques. The genus *Weissella* was named after the German microbiologist Norbert Weiss, known for his many contributions in the field of lactic acid bacteria research (Collins et al., 1993). Since the original description of the genus by Collins et al. (1993), various new species of *Weissella* have been described, so that currently the genus comprises 19 validated species (Figure 1). Key to these new species descriptions in the relevant studies were 16S rRNA gene sequence and DNA:DNA hybridization analyses, together with phenotypic data in a polyphasic taxonomical approach. Thus, the *Weissella* species grouped in five phylogenetic branches based on 16S phylogeny, with *W. soli, W. diestrammenae*, *W. koreensis, W. kandleri*, and *W. oryzae* as members of the first branch, *W. cibaria* and *W. confusa* as members of a second and *W. thailandensis, W. hellenica* and *W. paramesenteroides* occurring in a third branch. *W. ceti*, *W. halotolerans, W. viridescens, W. minor*, and *W. uvarum* are associated with the fourth branch, and *W. beninensis, W. fabalis, W. fabaria*, and *W. ghanensis* with the fifth (Figure 1). De Bruyne et al. (2010) showed that an improved phylogeny of *Weissella* based on *phe*S gene sequences was possible, due to the higher discriminatory power of this marker gene when compared to the 16S rRNA gene. Based on the *phe*S phylogenetic investigation (Figure 1), the authors showed that the new species described in that study as *W. fabaria*, together with the then already described *W. ghanensis* species clustered together as a first divergent line within the genus *Weissella*. Subsequent to the study of De Bruyne et al. (2010), two further novel species, i.e., *W. beninensis* and *W. fabalis* were described (Padonou et al., 2010; Snauwaert et al., 2013) that also grouped together with *W. fabaria* and *W. ghanensis* into a well-defined cluster. Thus, these four species appear to constitute this first divergent line of species within the genus *Weissella*. The term “species groups” has been previously used to group species that occur in phylogenetically closely related groups as in the case, e.g., for the enterococci (Švec and Franz, 2014). This has so far not been done for species occurring in the genus *Weissella*. Based on the clear grouping of species into 5 well-defined clusters, these groups could be designated as the *W. kandleri, W. confusa, W. halotolerans, W. paramesenteroides*, and *W. beninensis* species groups, respectively.

## General description of bacteria belonging to the genus *Weissella*

Bacteria belonging to the genus *Weissella* are Gram-positive, catalase-negative, non-endospore forming cells with coccoid or rod-shaped morphology (Collins et al., 1993; Björkroth et al., 2009, 2014). The *Weissella* species belong to the phylum *Firmicutes*, class *Bacilli*, order *Lactobacillales* and family *Leuconostocaceae* (Collins et al., 1993). Only *W. beninensis* was reported to be motile (Padonou et al., 2010), with all other species being non-motile. As in the original description of the genus *Weissella* (Collins et al., 1993), the bacteria of this genus were described to be non-motile, this motile characteristic of *W. beninensis* is not in accordance with the description of general characteristics of bacteria in this genus, and consequently the genus description was emended by Padonou et al. (2010) to account for atypical motility of this particular species. *Weissella* bacteria are facultatively anaerobic chemoorganotrophs with an obligately fermentative metabolism. They do not possess cytochromes and ferment glucose heterofermentatively via the hexose-monophosphate and phosphoketolase pathways. End products of glucose heterofermentation include lactic acid (with some species producing only d(−)and others both d(−) and l(+) lactic acid enantiomers), gas (CO_2_) and ethanol and/or acetate (Collins et al., 1993; Björkroth et al., 2014). The bacteria have complex nutritional requirements and need peptides, amino acids, fermentable carbohydrates, nucleic acids, fatty acids and vitamins for growth. All species grow at 15°C and some can grow up to 42–45°C. Production of dextran, hydrolysis of esculin and production of ammonia from arginine are variable characteristics for the different species, and may be used as phenotypic tests to aid in species identification (Table 1). The same applies for fermentation of sugars such as cellobiose, fructose, galactose, lactose, maltose, melibiose, raffinose, ribose, sucrose, trehalose and xylose (Table 1). The cell wall peptidoglycan is based on lysine as the diamino acid and, apart from *W. kandleri*, all contain alanine or alanine and serine in the interpeptide bridge (Björkroth et al., 2009). The interpeptide bridge of *W. kandleri* contains glycine (Holzapfel and van Wyk, 1982; Björkroth et al., 2009). The mol% G+C content of the different *Weissella* species ranges between 37 and 47%, which is close to the recommended 10% that should not be exceeded for genus definition (Stackebrandt and Liesack, 1993).

**Table 1:** **Differential characteristics of *Weissella* species**.

**Characteristic**	**1**	**2**	**3**	**4**	**5**	**6**	**7**	**8**	**9**	**10**	**11**	**12**	**13**	**14**	**15**	**16**	**17**	**18**	**19**
**ACID PRODUCED FROM**																			
Arabinose	−	−	+	−	+	−	−	−	−	+	−	+	−	+	d	+	+	−	−
Cellobiose	d	−	+	+	ND	+	+	+	−	−	−	−	+	−	d	−	−	−	−
Fructose	+	−	+	+	−	+	+	+	+	+	+	ND	+	+	+	−	+	+	+
Galactose	+	−	−	+	−	−	−	−	−	−	+	−	−	+	+	−	+	−	−
Maltose	+	+	+	+	+	+	−	+	+	+	−	−	+	+	+	+	+	−	+
Melibiose	+	−	−	−	ND	−	−	−	−	−	−	−	−	+	+	+	+	−	−
Raffinose	+	−	−	−	ND	−	−	−	−	−	−	−	−	−	d	+	+	−	−
Ribose	d	+	−	+	+	−	−	−	+	−	+	+	+	+	d	+	+	+	−
Sucrose	+	−	+	+	ND	−	−	d	−	+	−	−	+	−	+	+	d	−	d
Trehalose	d	+	−	−	ND	+	+	+	−	+	−	−	+	+	+	+	d	+	d
Xylose	−	−	+	+	+	−	−	−	−	−	−	+	−	−	d	+	−	−	−
Esculin hydrolysis	+	+	+	+	+	+	+	+	−	ND	−	−	+	−	v	+	−	−	−
Ammonia from arginine	+	v	+	+	+	+	+	+	+	−	+	+	+	+	−	+	−	+	−
Dextran formation	ND	−	+	+	ND	+	+	+	ND	−	+	+	−	−	−	−	−	−	ND
Lactic acid configuration	dl	dl	dl	dl	d	d	dl	dl	dl	d	dl	d	dl	d	d	d	d	d	dl
Mol% G+C content	37	39.2	44–45	45–47	45	37	38	40	44	39–40	39	37	44	40.6	37–38	43	38–41	39.1	41–44

*Weissella species indicated as follows: 1, W. beninensis; 2, W. ceti; 3, W. cibaria; 4, W. confusa; 5, W. diestrammenae; 6, W. fabalis; 7, W. fabaria; 8, W. ghanensis; 9, W. halotolerans; 10, W. hellenica; 11, W. kandleri; 12, W. koreensis; 13, W. minor; 14, W. oryzae; 15, W. paramesenteroides; 16, W. soli; 17, W. thailandensis; 18, W. uvarum; 19, W. viridescens*.

*+, 90% or more strains are positive; -, 90% or more strains negative; d, 11–89% of strains positive; ND, no data available; v, variable*.

*Data partially adapted from Collins et al. (1993); Tanasupawat et al. (2000); Björkroth et al. (2002); Magnusson et al. (2002); De Bruyne et al. (2008, 2010); Vela et al. (2011); Oh et al. (2013); Snauwaert et al. (2013); Tohno et al. (2013) and Nisiotou et al. (2014)*.

## Ecology

Bacteria of the genus *Weissella* inhabit a variety of ecological niches, including soil (mainly *W. soli*) (Magnusson et al., 2002; Chen et al., 2005), sludge of milking machines (*W. minor*) (Kandler et al., 1983), sediments of a coastal marsh (*W. cibaria* and *W. confusa*) (Zamudio-Maya et al., 2008), sediments and fish from the Bahía Blanca estuary (*W. viridescens*) (Sica et al., 2010) and lake water (*W. cibaria*) (Yanagida et al., 2007), plants (Table 2), a huge variety of fermented foods (Table 3), the oral cavity, breast milk, the uro-genital and gastro-intestinal tracts of humans (Table 4), as well as the skin, milk and gastro-intestinal tract of many animals (Table 5).

**Table 2:** **Occurrence of *Weissella* species in different environmental habitats**.

**Species**	**Habitat or Source**	**References**
*W. cibaria*	Japanese horseradish, orange, pineapple, banana, chili bo	Endo et al., 2009
	Tomatoes	Di Cagno et al., 2009a
	Fluted pumpkin vegetable (*Telfairia occidentalis*) and green vegetable (*Amaranthus spinosus*)	Emerenini et al., 2014
	Bee pollen	Belhadj et al., 2014
	Wheat flour	Alfonzo et al., 2013
	Corn stovers	Pang et al., 2011
	Blackberries	Di Cagno et al., 2011
	Papaya	Di Cagno et al., 2011
*W. confusa*	Rhizosphere of olive trees, soil surrounding rhizospere	Fhoula et al., 2013
	Raw red and yellow pepper	Di Cagno et al., 2009b
	Heroin	Cho et al., 2014
	Sugar cane and carrot juice	Hammes and Vogel, 1995
*W. halotolerans*	Rhizosphere of olive tree, soil surrounding rhizospere	Fhoula et al., 2013
*W. hellenica*	Vegetative forage crops (mixed pasture of timothy and orchardgrass)	Tohno et al., 2012
*W. hellenica*	Heroin	Cho et al., 2014
*W. kandleri*	Desert spring and desert plants	Holzapfel and van Wyk, 1982
*W. kimchii (W. cibaria)*	Fluted pumpkin vegetable (*Telfairia occidentalis*) and green vegetable (*Amaranthus spinosus*)	Emerenini et al., 2014
*W. paramesenteroides*	Rhizosphere of olive trees	Fhoula et al., 2013
	Fluted pumpkin vegetable (*Telfairia occidentalis*) and green vegetable (*Amaranthus spinosus*)	Emerenini et al., 2014
	Chardonnay grapes, Semillon and Sauvignon Blanc grapes	Bae et al., 2006
	Indian goosegrass	Pang et al., 2012
	Vegetative forage crops (mixed pasture of timothy and orchardgrass)	Tohno et al., 2012
*W. soli*	Carrots	Di Cagno et al., 2008

**Table 3:** **Isolation of *Weissella* species from (fermented) foods**.

**Species**	**Food source**	**Detection method**	**Country**	**References**
*Weissella* spp.	Joetgal (fermented sea food)	Pyrosequencing (culture-independent)	Korea	Roh et al., 2010
*Weissella* spp., *W. paramesenteroides*	Mexican pozol (fermented maize dough)	Culture-independent PCR-DGGE	Mexico	Ampe et al., 1999
*W. cibaria, W. soli, W. koreensis*	Dongchimi, (watery kimchi)	Pyrosequencing (culture-independent)	Korea	Jeong et al., 2013
*Weissella* spp. *W. soli, W. beninensis*	Malt (produced by industrial malting)	Culture-independent T-RFLP (terminal restriction fragment length polymorphism) and pyrosequencing	Belgium	Justé et al., 2014
*W. confusa, W. oryzae*	Unpasteurized Boza (ceral-based fermented beverage)	Culture-dependent 16S rRNA gene sequencing and culture-independent PCR-DGGE	Bulgaria	Osimani et al., 2015
*W. hellenica, W. paramesenteroides*	Raw milk cheeses	Pyrosequencing of DNA and cDNA	Denmark	Masoud et al., 2012
*W. confusa, W. cibaria*	Sourdough	Physiological and biochemical tests	France	Bounaix et al., 2010
*W. cibaria, W. soli, W. minor, W. viridescens*	Sorghum silage	Lab-made biochemical tests	Algeria	Chahrour et al., 2013
*W. confusa*	Wheat sourdough	Culture dependent 16S rRNA sequencing	Italy	Corsetti et al., 2001
	Cheese, Nono	Culture dependent partial 16S rRNA gene sequencing	Nigeria	Ayeni et al., 2011
	Masai fermented milk	Physiological and biochemical tests	Northern Tanzania	Isono et al., 1994
	Chili bo (Malaysian food ingredient)	Culture dependent: biochemical tests and 16S rRNA sequencing	Malaysia	Leisner et al., 1999
	Suusac (fermented camel milk)	Culture-dependent 16S rRNA gene sequencing	Africa	Jansa et al., 2012
	Emmer and spelt flour for bread making	Culture-dependent partial sequencing of recA, 16S/23S rRNA spacer region and pheS genes.	Italy	Coda et al., 2010, 2014
	Cauliflower and mixed-vegetable spontaneous fermentation	Culture-dependent (GTG)5-PCR fingerprinting, 16S rRNA gene sequencing and culture-independent PCR-DGGE	Romania	Wouters et al., 2013
	Togwa (Tanzanian fermented food)	Physiological and biochemical tests	Tanzania	Mugula et al., 2003
	Douchi (salt-fermented soybean food)	Culture-dependent partial 16S rRNA sequencing	China	Liu et al., 2012
	Bushera (fermented beverage)	Physiological and biochemical tests	Uganda	Muyanja et al., 2003
	Stinky tofu (fermented tofu)	Culture-dependent 16S rRNA gene sequencing	Taiwan	Chao et al., 2008
	Kulenaoto (fermented milk)	Physiological and biochemical tests	Kenya	Mathara et al., 2004
	Doenjang (fermented soybean paste)	Culture-independent PCR-DGGE	Korea	Kim et al., 2009
	NtobaMbodi (fermented cassava leaves)	Culture-dependent partial 16S rRNA gene sequencing	Congo	Ouoba et al., 2010
	Wheat sourdough	Culture-dependent partial 16S rRNA gene sequencing	France	Robert et al., 2009
	Zichi (Sardinian sourdoughbread)	Culture-dependent 16S rRNA gene sequencing	Italy	Catzeddu et al., 2006
	Kimchi	Culture-independent PCR-DGGE	Korea	Lee et al., 2005
	Wheat sourdough	Culture-dependent partial 16S rRNA gene sequencing	France	Robert et al., 2009
*W. cibaria*	Greek Traditional Wheat Sourdoughs	Culture-dependent DNA-DNA hybridization, and 16S ribosomal DNA sequence analysis	Greece	De Vuyst et al., 2002
	Buckwheat and teff sourdoughs (spontaneously fermented)	Culture-dependent 16S rRNA gene sequencing and Culture-independent PCR-DGGE	Ireland	Moroni et al., 2011
	Traditional Belgian sourdoughs	Culture-depedent 16S rRNA gene sequencing, DNA-DNA hybridization, REP-PCR and phenylalanyl-tRNA synthase (*pheS*) gene sequence analysis	Belgium	Scheirlinck et al., 2007
	Nukadoko (naturally fermented rice bran mash used for pickling vegetables)	Pyrosequencing (culture-independent)	Japan	Ono et al., 2014
	Buchwheat and teff sourdoughs	Culture-dependent partial 16S rRNA sequencing	Ireland	Moroni et al., 2011
	Fermented Jalapeño pepper	Culture-dependent partial 16S rRNA sequencing	Mexixo	González-Quijano et al., 2014
	Douchi (salt-fermented soybean food)	Culture-dependent partial 16S rRNA sequencing	China	Liu et al., 2012
	Cassava	Physiological and biochemicaltests; culture-dependent partial 16S rRNA sequencing	South Africa, Benin, Kenya,Germany	Kostinek et al., 2007
	Plaa-som (Thai fermented fish product)	Biochemicaltests; culture-dependent partial 16S rRNA sequencing	Thailand	Srionnual et al., 2007
	Yan-dong-gua (fermented waxgourd)	Physiological analysis; culture-dependent RFLP and partial 16S rRNA sequencing	Taiwan	Lan et al., 2009
	Stinky tofu	Culture-dependent 16S rRNA gene sequencing	Taiwan	Chao et al., 2008
	Pickles		China	Zhao et al., 2008
	Fu-tsai (fermented mustard)	Physiological analysis; culture-dependent RFLP and partial 16S rRNA sequencing	Taiwan	Chao et al., 2009
	Yan-jiang (fermented ginger)	Culture-dependent 16S rRNA gene sequencing	Taiwan	Chang et al., 2011
	Tarhana (yogurt and wheat flour-based fermented food)	Culture-dependent sequencing of the 16S rRNA	Turkey	Sengun et al., 2009
	Thai fermented pork sausage	Culture-dependent biochemical tests and partial 16S rRNA gene sequencing	Japan	Thongsanit et al., 2009
	Jiang-gua (fermented cucumbers)	Culture-dependent RFLP partial sequencing of the 16S rRNA	Taiwan	Chen et al., 2012
	Plaa-som (fermented fish)	Culture-dependent ARDRA and partial sequencing of the 16S rRNA	Thailand	Kopermsuba and Yunchalard, 2010
*W. ghanensis*	Ghanaian cocoa beans fermentation	Culture-dependent 16S rRNA gene sequencing, physiological and biochemical tests	Ghana	De Bruyne et al., 2008
*W. kandleri*	Koumiss	Physiological and biochemicaltests; culture-dependent 16S rRNA gene sequencing	Mongolia	Wu et al., 2009
*W. kimchi* (re-classified as *W. cibaria* by Ennahar and Cai, 2004)	Kimchi (fermented cabbage)	Culture-dependent 16S rRNA gene sequencing	Korea	Choi et al., 2002
	Cauliflower and mixed-vegetable spontaneous fermentation	Culture-dependent (GTG)_5_-PCR fingerprinting, 16S rRNA gene sequencing	Romania	Wouters et al., 2013
*W. koreensis*	Kimchi	Culture-dependent morphological, physiological and chemotaxonomic tests, 16S rRNA gene sequencing	Korea	Lee et al., 2002
	Kimchi	Culture-independent 16S rRNA gene clone libraries	Korea	Park et al., 2010
	Kimchi	Culture-dependent physiological tests, partial 16S rRNA gene sequencing and restriction enzyme analysis	Korea	Cho et al., 2006
*W. halotolerans*	Fermented sausage	Not specified	Portugal	Pereira et al., 2009
*W. hellenica*	Naturally fermented sausage	Physiological and biochemicaltests	Greece	Samelis et al., 1994
	Jiang-gua (fermented cucumbers)	Culture-dependent RFLP partial sequencing of the 16S rRNA	Taiwan	Chen et al., 2012
	Croatian raw ewe's milk cheeses	Pyroseqeuncing (culture-independent)	Croatia	Fuka et al., 2013
	Fermented sausage	Culture-dependent PCR-DGGE and 16S rRNA gene sequencing	Italy	Urso et al., 2006
	Sausage	Culture-dependent PCR-DGGE and partial 16S rRNA gene sequencing	Italy	Cocolin et al., 2009
*W. minor*	Gari (fermented cassava)	Physiological and biochemical tests; culture-dependent partial 16S rRNA sequencing	Africa	Kostinek et al., 2005
	sludge of milking machines	16S rRNA gene sequencing, DNA-DNA hybridization	Germany	Kandler et al., 1983
*W. paramesenteroides*	Fermented sausage	Culture-dependent PCR-DGGE and 16S rRNA gene sequencing	Italy	Urso et al., 2006
	Goat's milk cheese	API 50 CH and API 20 STREP systems (BioMerieux)	Spain	Mas et al., 2002
	Joetgal (fermented sea food)	Culture-independent PCR-DGGE	Korea	Roh et al., 2010
	Nukadoko (naturally fermented rice bran mash used for pickling vegetables)	Pyrosequencing (culture-independent)	Japan	Ono et al., 2014
	Douchi (salt-fermented soybean food)	Culture-dependent partial 16S rRNA sequencing	China	Liu et al., 2012
	Croatian raw ewe's milk cheeses	Pyrosequencing (culture-independent)	Croatia	Fuka et al., 2013
	Yan-dong-gua (fermented waxgourd)	Culture-dependent PCR-DGGE and 16S rRNA gene sequencing	Taiwan	Lan et al., 2009
	Stinky tofu	Culture-dependent PCR-DGGE and partial 16S rRNA gene sequencing	Taiwan	Chao et al., 2008
	Fu-tsai	Culture-dependent partial sequencing of the 16S rRNA, *rpo*A, *phe*S and *dna*A genes	Taiwan	Chao et al., 2009
	Cassava	Culture-dependent physiological and biochemical tests, partial 16S rRNA gene sequencing	South Africa, Benin, Kenya, Germany	Kostinek et al., 2007
*W. soli*	Stinky tofu	Culture-dependent phenotypic and chemotaxonomic tests, partial 16S rRNA gene sequencing	Taiwan	Chao et al., 2008
*W. taj-apis^a^*	Honey	16S rRNA gene sequencing	Malaysia	Tajabadi et al., 2012
*W. thailandensis*	Pla-ra and pla-chom (fermented fish)	Phenotypic and chemotaxonomic tests; culture-dependent partial 16S	Thailand	Tanasupawat et al., 1998, 2000
*W. viridescens*	Dry-fermented sausage		Greece	Papamanoli et al., 2003
	Cauliflower and mixed-vegetable spontaneous fermentation	Culture-dependent (GTG)5-PCR fingerprinting, 16S rRNA gene sequencing	Romania	Wouters et al., 2013
	Nham (Thai-fermented pork sausage)	Culture-dependent physiological and biochemical tests, 16S rRNA gene sequencing	Thailand	Pringsulaka et al., 2011
	Doenjang (fermented soy bean paste)	Culture-independent PCR-DGGE	Korea	Kim et al., 2009
*W. uvarum*	Wine grapes	16S rRNA gene sequencing, DNA-DNA hybridization	Greece	Nisiotou et al., 2014

a *Weissella taj-apisdescribed by Tajabadi et al. (2012) is currently an unvalidated species description*.

**Table 4:** ***Weissella* species in saliva, feces and vagina of humans**.

**Species**	**Habitat or Source**	**References**
*W. cibaria*	Children's saliva (4–7 years old)	Kang et al., 2006a,b
	Human saliva	Kang et al., 2011
	Human feces	Wang et al., 2008
		Nistal et al., 2012
	Human vagina	Nam et al., 2007
*W. confusa*	Human faecis	Ponnusamy et al., 2011
	Human feces (adults, mothers and babies)	Albesharat et al., 2011
	Human feces	Zhang et al., 2014
		Walter et al., 2001
*W. confusa* and *W. Cibaria*	Human feces	Gomathi et al., 2014
		Chun et al., 2007; Lee et al., 2012
	Breast milk, vaginal swab and infant feces	Martín et al., 2007a,b
*W. kimchii (W. cibaria)*	Human vagina	Lee, 2005
	Human vagina	Jin et al., 2007
*W. paramesenteroides*	Feces of breast-fed infants	Rubio et al., 2014
*W. viridescens*	Human vagina	Silvester and Dicks, 2003
		Jin et al., 2007

**Table 5:** ***Weissella* species in healthy animals' milk and skin**.

**Species**	**Habitat or Source**	**References**
*Weissella* spp.	Ewe's milk	Aquilanti et al., 2007
	Ileal digesta of piglets fed diets supplemented with 200 or 3000 ppm ZnO	Vahjen et al., 2010
*W. cibaria*	Camel's milk	Merzouk et al., 2013
	Goat's milk	Elavarasi et al., 2014
	Feces of individually (healthy) owned dogs	Graef et al., 2005
	Acquatic animals	Muñoz-Atienza et al., 2013
	Feces of farmed Atlantic salmon (*Salmo salar* L.)	Hovda et al., 2012
	Gastro-intestinal tract of brown trout	Abid et al., 2013
	Human feces and human gall, Canary liver	Björkroth et al., 2002
*W. confusa*	Cow's milk	Zambou et al., 2008
	Cow's intestine	Ayeni et al., 2011
	Intestines of adult farmed seabass (*Lates calcarifer*)	Sirirat et al., 2008
	Intestines of farmed Asian seabass (*Lates calcarifer*)	Rengpipat et al., 2008
*W. confusa* and *W. cibaria*	Canine feces	Beasley et al., 2006
*W. diestrammenae*	Gut of a camel cricket	Oh et al., 2013
*W. hellenica*	Cow's milk	Masoud et al., 2012
	Intestinal contents of flounder (*Paralichthys olivaceus*)	Cai et al., 1998
*W. paramesenteroides*	Cow's milk	Espeche et al., 2009
	Distal gut contents of rainbow trout fed different plant based diets	Desai et al., 2012
*W. viridescens*	Canine milk	Martín et al., 2010
*W. paramesentroides*	Midgut of *Ostrinia nubilalis*	Belda et al., 2011

Typical for lactic acid bacteria, to which the *Weissella* spp. belong, is their association with and adaptation to nutrient rich habitats (Makarova et al., 2006) including various food sources. Some *Weissella* species, i.e., *W. viridescens*, *W. halotolerans*, and *W. hellenica*, are mainly associated with meat and meat products, and were reported as part of the predominant microbiota responsible for quality fluctuations of packaged and chill-stored food products (Pothakos et al., 2014). Similarly, *W. halotolerans* has been reported to predominate in the microbial spoilage population of vacuum-packaged, charcoal-broiled European river lamprey (*Lampetra fluviatilis*) (Merivirta et al., 2005). *W. viridescens* causes spoilage of cured meats due to a green discoloration (Niven and Evans, 1957) and is involved in spoilage of the Spanish blood sausage Morcilla de Burgos (Santos et al., 2005; Koort et al., 2006; Diez et al., 2009) and of vacuum-packaged cooked sausages (Korkeala and Björkroth, 1997; Iacumin et al., 2014).

*Weissella* species are also commonly found in habitats associated with the human or animal body, e.g., the gastrointestinal tract or in human breast milk. *W. cibaria* was found to be present in all fecal samples from healthy adults, but less frequent in the fecal samples of celiac disease patients (Nistal et al., 2012). *W. confusa* was shown to be more widely distributed in the feces of non-irritable bowel syndrome patients than in the feces of patients affected by this disease (Ponnusamy et al., 2011). High loads of *Weissella* spp. were also found in the ileal microbiota of piglets fed with different amounts of zinc oxide (ZnO), an amphoteric molecule that is widely used as feed additive for the prophylaxis of diarrhea in piglets (Vahjen et al., 2010). *W. confusa* was found in the breast milk, as well as in the feces of both mothers and infants (Martín et al., 2007a,a; Albesharat et al., 2011), confirming the hypothesized mechanism of vertical transfer from the mother's gut to the corresponding milk and subsequently from the milk to the infant's gut. Regarding the presence of *Weissella* and other microorganisms in human milk, possible mechanisms by which these bacteria can reach the mammary gland (i.e., either by contamination or by active migration) have been reviewed recently (Jeurink et al., 2013). It has been suggested (Lahtinen et al., 2012) that *Weissella* strains from human milk, stem from an environmental source (e.g., soil, vegetation). Indeed, the mode of delivery at birth, the kind of diet, as well as the health status of humans and animals may affect the composition of the microbiota of the oral cavity, the gastro-intestinal and uro-genital tracts. In agreement with this, by using a high-throughput sequencing approach, Belda et al. (2011) could show that *W. paramesenteroides* occurs at notably higher levels in the midgut of lab-reared populations of the European Corn Borer *Ostrinia nubilalis* than in the field population, in which gram-negative species were found to predominate. This probably was the result of an increase in cell numbers due to the multiplication of these bacteria in the artificial diet prior to insect feeding. This finding led to the hypothesis that food exerts a selection pressure on the intestinal microbiota (Belda et al., 2011).

## *Weissella* strains associated with human clinical infections

*Weissella* strains have been isolated from clinical specimens such as blood, skin, infected wounds and feces of both humans and animals (Table 6). Apart from Kulwichit et al. (2007), who identified a *Weissella* strain from the blood of a patient as *W. viridescens*, and others from urine, lung swabs and blood of patients with bacteremia as *W. cibaria*, the only species of *Weissella*, which have been described as opportunistic pathogens of humans or as emerging pathogen for farmed rainbow trouts are *W. confusa* and *W. ceti*, respectively. In particular, *W. confusa* was isolated from several human and clinical specimens in cases of polymicrobial infections (Green et al., 1990, 1991; Riebel and Washington, 1990; Bantar et al., 1991; Olano et al., 2001; Björkroth et al., 2002). The isolation of the strains from polymicrobial infections did not allow an unequivocal clinical significance of this species. Subsequently, however, this species was also described as sole microbial agent in various infections which allowed the description of *W. confusa* as an opportunistic pathogen. Indeed, *W. confusa* was the causative agent of infections such as a systemic infection in a mona monkey (*Cercopithecus mona*) (Vela et al., 2003), a fatal case of endocarditis (Flaherty et al., 2003), a severe infective endocarditis of native valves (Shin et al., 2007), a postoperative osteomyelitis with chronic discharge in a young female (Kulwichit et al., 2008), and a sepsis in a 48-year-old male who was operated for adenocarcinoma of the gastro-oesophageal junction and who was maintained on a total parenteral nutrition (Kumar et al., 2011). Furthermore, it also caused infection in patients with hepatocellular carcinoma occurring after liver transplant (Harlan et al., 2011), in patients with acute lymphocytic leukemia undergoing autologous stem cell transplantion (Salimnia et al., 2011), and in a patient with a prostetic joint infection (Medford et al., 2014). A large case series (i.e., a descriptive study that follows a group of patients who have a similar diagnosis or who are undergoing the same procedure over a certain period of time; http://jbjs.org/content/91/Supplement_3/21), was reported by Lee et al. (2011) and involved 10 patients with bacteremia. Risk factors for invasive infection in this group included a central line catheter insertion, a concurrent polymicrobial bacteremia and an immunocompromised host, together with gastrointestinal manipulation through endoscopy, or surgery that may have allowed the contamination of *Weissella* into the blood stream. Indeed, as highlighted by Medford et al. (2014), most cases of clinical infection with *Weissella* were associated with medical procedures within the period of infection. *W. confusa* was also found to cause neonatal sepsis in a foal (Lawhon et al., 2014). *W. ceti* has recently been recognized as the etiological agent of the so-called “weissellosis” (Welch et al., 2014), an emergent disease occurring in farmed rainbow trout (*Oncorhynchus mykiss*) causing septicemia with a high mortality rate (Costa et al., 2015). Weissellosis outbreaks have been reported from commercial trout farms in the United States, China and Brazil (Liu et al., 2009; Figueiredo et al., 2012; Welch and Good, 2013; Costa et al., 2015). Symptoms of this disease include lethargy and anorexia, extensive ocular lesions, occasional cerebral hemorrhage and dark skin coloration (Welch et al., 2014). Apparently, high summer temperatures seems to be the main predisposing factor for this emerging disease that appears to affect only the large-size fishes (0.5–1 kg) in a trout farm, while ongoing studies are focusing on ascertaining the pathogen's route of infection and its reservoirs (Welch et al., 2014).

**Table 6:** ***Weissella* species from human and animal clinical specimens**.

**Species**	**Source**	**References**
*W. ceti*	Diseased beaked whales (*Mesoplodon bidens*) (muscle tissue, brain, kidney, lymph nodes, spleen of four different animals).	Vela et al., 2011
	diseased rainbow trout (*Oncorhynchus mykiss*)	Liu et al., 2009; Figueiredo et al., 2012
*W. cibaria*	Dog ear (otitis)	Björkroth et al., 2002
	Human blood (bacteremia)	Kulwichit et al., 2007
	Human lung swab (bacteremia)	Kulwichit et al., 2007
	Human urine	Kulwichit et al., 2007
*W. confusa*	Blood of a neonatal foal with septicemia	Lawhon et al., 2014
	Intestine, lung, liver, and brain of a female mona monkey (*Cercopithecus mona*) with systemic infection	Vela et al., 2003
	Human feces, Human gall, Human drain, necropsy specimens of a dog	Björkroth et al., 2002
	Human blood cultures (of patients with bacteremia)	Olano et al., 2001
	Human feces (children)	Green et al., 1990
	Human feces (of pediatric liver transplant recipients)	Green et al., 1991
	Human peritoneal fluids (after hemicolectomy) and abdominal walls of two patients	Riebel and Washington, 1990
	Human blood (of patients with monomicrobial bacteremia)	Kumar et al., 2011
	Human blood (in an immune competent patient with underlying intramural hematomas of the aorta)	Lee et al., 2013
	Human blood of 10 patients with bacteremia	Lee et al., 2011
	Human blood cultures (of patients with infective endocarditis)	Flaherty et al., 2003, Shin et al., 2007
	Purulent material from the thumb abscess of human	Bantar et al., 1991
	Human blood (bacteremia)	Harlan et al., 2011
	Human blood (bacteremia)	Kulwichit et al., 2007
	Human bone (osteomyelitis)	Kulwichit et al., 2007
	Human blood from patient with bacteremia	Salimnia et al., 2011
	Human blood of two patients with bacteremia	Fairfax et al., 2014
	Human aspirate from a knee with prosthetic joint	Medford et al., 2014
*W. viridescens*	Fecal DNA from celiac children	Sanz et al., 2007
	Human blood (bacteremia)	Kulwichit et al., 2007
*Weissella* spp.	Human feces of children diagnosed with human immunodeficiency virus (HIV)	Dicks et al., 2009

As suggested by several authors (Lahtinen et al., 2012; Fairfax et al., 2014; Medford et al., 2014), infections caused by *Weissella*, as those caused by *Leuconostoc*, are mainly due to their natural vancomycin resistance, and usually occur in cases of immunosuppression or underlying disease of the host. However, infections caused by *Weissella* spp. are generally rare, although an underestimation may occur as a result of the inability of commercial bacterial identification systems [such as the API 50 CHL kit (BioMérieux, Lyon, France) etc.] in identifying these bacteria as they closely resemble viridans streptococci (Fairfax et al., 2014).

## Potentially probiotic or technologically uses of *Weissella* strains

In several studies, *Weissella* strains were screened for antimicrobial activity (Nam et al., 2002; Pal et al., 2010; Ndagano et al., 2011; Papagianni and Papamichael, 2011; Masuda et al., 2012; Papagianni, 2012; Vitali et al., 2012; Leong et al., 2013; Papagianni and Sergelidis, 2013; Serna-Cock et al., 2013; Yoshiyama et al., 2013; Emerenini et al., 2014). Six bacteriocins have so far been reported for *Weissella* strains belonging to the *W. cibaria*, *W. paramesenteroides*, and *W. hellenica* species (Table 7). Among these, the listericidal bacteriocin weissellin A was further investigated for its technological application in fermented sausages (Papagianni, 2012; Papagianni and Papamichael, 2012; Papagianni and Sergelidis, 2013), while the bacteriocinogenic *W. hellenica* strain D1501 was successfully used to enhance the shelf-life of tofu (Chen et al., 2014b).

**Table 7:** **Bacteriocinogenic *Weissella* strains, class, name, organisms against which the bacteriocins were active and relevant reference**.

**Name**	**Class**	**Producer organisms**	**Sensitive indicator strains**	**References**
Weissellicin 110	Unclassified	*W. cibaria* 110	*Lactobacillus sakei* JCM 1157*, L. sanfranciscensis* JCM 5668*, L. homohiochii* JCM 1199*, L. coryniformis* subsp. *coryniformis*, JCM 1164, *L. acetotolerans JCM 3825—Weissella halotolerans* JCM1114, *W. kandleri* JCM 5817, *W. paramesenteroides* JCM 9890, *Leuconostoc lactis* JCM 6123	Srionnual et al., 2007
Weissellin A	Class IIA	*W. paramesenteroides* DX	*Bacillus cereus* LMG13569, *Clostridium sporogenes* NCTC533, *C. thiaminolyticum* ATCC15579, *Enterococcus faecalis* NCTC8176*, Lactobacillus brevis* ATCC8287, *L. bulgaricus* LMG13551, *L. casei* ATCC344*, L. curvatus* ATCC51436, *L. jensenii* ATCC25258, *L. plantarum* CECT220, L. sakei CECT906T*, Lactococcus lactis* LM0230, *Lact. lactis* ATCC11454*, Lact. lactis* IL1403*, Lact. lactis* subsp. *cremoris* MC1363, *Leuconostoc mesenteroides* ATCC19254*, Listeria innocua* ATCC BAA-680D*, List. monocytogenes* ATCC19111*, Micrococcus luteus* CECT241, *Pediococcus acidilactici* ATCC25740, *P. pentosaceus* ATCC 33316, *P. pentosaceus* LMG13560, *Staphylococcus carnosus* LMG13564	Papagianni and Papamichael, 2011
Weissellicin L	Unclassified	*W. hellenica* 4-7	*L. monocytogenes* ATCC 19111, *L. sakei* subsp. *sakei* JCM 1157, *L. bulgaricus* ATCC 11842, *W. paramesenteroides* ATCC33313, *W: hellenica* ATCC 51523, *W. viridescens* ATCC 12706, *S. thermophilus* ATCC 19258	Leong et al., 2013
Weissellicin D	Unclassified	*W. hellenica* D1501	*L. lactis* ssp. *lactis*, *Lactobacillus fermentum* ATCC 14931, *Lb. sake*, *Lb. plantarum* 70810, *Lb. bulgaricus* ATCC 7830, *Lb. helveticus* Mb2-1, L*b. paracasei, Lb. curvatus, Lb. brevis, Pediococcus pentosaceus* CGMCC1.2695, *Streptococcus thermophilus* CGMCC1.6472*, Staphylococcus aureus* ATCC 6538*, Bacillus subtilis* ATCC 6633, *B. cereus* ATCC 11778, *Pseudomonas aeruginosa, Listeria monocytogenes* CMCC 54004, *Micrococcus luteus* CMCC28001, *Saccharomyces cerevisiae* ATCC 26603, *Debaromyces hansenii* ATCC 4143, *Kluyveromyces marxianus, Candida albicans* CMCC 28001, *Mucor* CICC 2521	Chen et al., 2014a
Weissellicin M Weissellicin Y	Unclassified Unclassified	*W. hellenica* QU 13	*^a^L. lactis* ssp. *lactis* ATCC 19435T*, L. lactis* ssp. *lactis* NCDO 497, *Lactobacillus sakei* ssp. *sakei* JCM 1157T, *Lb. plantarum* JCM 1149T, *Weissella cibaria* JCM 12495T, *W. hellenica* JCM 10103T, *W. paramesenteroides* JCM 9890T, *W. confusa* JCM 1093T, *Pediococcus pentosaceus* JCM 5885*, P. dextrinicus* JCM 5887T, *P. acidilactici* JCM 8797T, *Enterococcus faecium* JCM 5804T, *E. durans* NBRC 100479T, *E. faecalis* JCM 5803T, *Streptococcus bovis* JCM 5802T*, Str. dysgalactiae* ssp. *dysgalactiae* JCM 5673, *Bacillus coagulans* JCM 2257T, *B. circulans* JCM 2504T*, B. subtilis* ssp. *subtilis* JCM 1465T, *B. cereus* JCM 2152T, *Kocuria rhizophila* NBRC 12708, *Listeria innocua* ATCC 33090T, *Leuconostoc mesenteroides* ssp. *mesenteroides* JCM 6124T	Masuda et al., 2012

a *Both bacteriocins were active against these bacteria but weissellicin Y showed an overall weaker activity than weissellicin M*.

Aiming at developing novel probiotic foods or probiotic animal feeds, many researchers have isolated and screened *Weissella* strains from humans (Ayeni et al., 2011; Lee et al., 2012; Gomathi et al., 2014; Zhang et al., 2014), animal feces (Cai et al., 1998; Beasley et al., 2006; Muñoz-Atienza et al., 2013) as well as from a variety of food matrices, including vegetable, fruits, cured meat and dairy matrices, for their probiotic potential (Papamanoli et al., 2003; Vitali et al., 2012; Patel et al., 2013; Yoshiyama et al., 2013; Yang et al., 2014). However, only few studies investigated the probiotic potential of *Weissella* strains using *in vivo* studies. Wang et al. (2011) demonstrated that dietary supplementation with fermented garlic together with *W. koorensis* in growing pigs can improve the average daily gain and has a positive impact on the immune response during an inflammatory challenge (Wang et al., 2011). *W. cibaria* isolates from children's saliva were shown to inhibit *in vitro* biofilm formation and proliferation of one of the main bacterial pathogens in dental caries, especially in early-childhood caries, namely *Streptococcus mutans* (Kang et al., 2005). This inhibition occurred via the water soluble-polymers produced from sucrose by *Weissella*. Moreover, using an *in vivo* study on 72 volunteers who rinsed their teeth after brushing in the morning, afternoon and evening, with a rinse that contained the potential probiotic *W. cibaria* strain, a significant 20% reduction in plaque scores could be achieved. This indicated a high potential of *W. cibaria* isolates to inhibit biofilm formation (Kang et al., 2005). Hydrogen peroxide-producing weissellas, belonging to the *W. cibaria* species, were also isolated from children's saliva and were capable of inhibiting the *in vitro* production of halitosis indicators such as volatile sulfur compounds (VSC) produced by *Fusobacterium nucleatum*. Furthermore, these bacteria could inhibit the proliferation of five periodontopathic bacteria, including *F. nucleatum*. Moreover, clinical studies based on gargling with the best performing *W. cibaria* isolate resulted in a significant *in vivo* reduction of the level of VSC (Kang et al., 2006a,b). An *in vitro* antiflammatory activity of *W. cibaria*, which consisted of inhibition of interleukin (IL)-6 and IL-8 production from human mouth epithelial cells that were originally elicited by *F. nucleatum*, could also be demonstrated *in vitro*, highlighting once again the high probiotic potential of *W. cibaria* in controlling periodontal disease (Kang et al., 2011). For all these reasons, Kang et al. (2012) successfully investigated the stability of probiotic chewing gum containing a *W. cibaria* strain.

Moon et al. (2012) demonstrated in an *in vitro* study that intracellular lipid accumulation in 3T3-L1 cells could be inhibited by the ornithine rich cytoplasmic extract of *W. koreensis* OK1-6. Lately, it was demonstrated that kimchi fermented with this *W. koreensis* strain as starter culture has an anti-obesity effect in high-fat diet-induced obese mice (Park et al., 2012).

Nevertheless, it should be considered that the current legislation on probiotics and probiotic foods/feed is very different worldwide, with a stricter regulatory framework in the European Community. Indeed, the Panel on Dietetic Products, Nutrition and Allergies of the European Food Safety Authority has rejected more than 300 health claims on the benefits of probiotic bacteria, resulting in not one of the probiotic products being allowed to claim a health benefit for the strains they contain. As a consequence, in Europe not a single probiotic product, food or supplement, can mention the health benefits of the strains it includes. Moreover, considering that the most current and accepted definition (the FAO/WHO panel definition) of probiotics define them as “live microorganisms which when administered in adequate amounts confer a health benefit on the host” (Hill et al., 2014), even the word “probiotic” would not be allowed in the definition. These findings, together with the role as opportunistic pathogens of some weissellas and the intrinsic resistance to vancomycin and other antibiotics (Ouoba et al., 2008; Ayeni et al., 2011; Muñoz-Atienza et al., 2013), may drastically reduce the potential use of these bacteria as probiotics or even only as pro-technological (starter) bacteria in food, feed and supplements. Therefore, before thinking about using a *Weissella* strain for biotechnological and probiotic purposes, a thorough, strain-specific safety assessment would be mandatory.

## EPS and prebiotics producing strains

The ability to produce dextran is one of the distinctive phenotypic features of the genus *Weissella* (Collins et al., 1993; Björkroth and Holzapfel, 2006). In particular, strains of *W. confusa* and *W. cibaria* have received high attention in the last decade due to their ability to produce significant amounts of dextran (De Bruyne et al., 2008, 2010; Maina et al., 2008, 2011, 2013, 2014; Björkroth et al., 2009; Katina et al., 2009; Bounaix et al., 2010; Padonou et al., 2010; Ahmed et al., 2012; Amari et al., 2013; Bejar et al., 2013; Rao and Goyal, 2013a,b; Shukla et al., 2014; Wolter et al., 2014; Tingirikari et al., 2014a,b; Malang et al., 2015), fructan and heteropolysaccharides (Tieking et al., 2003; Di Cagno et al., 2006; Malik et al., 2009; Malik, 2012; Malang et al., 2015), and novel non-digestible oligosaccharides (Chun et al., 2007; Kang et al., 2009; Patel et al., 2013; Immerzeel et al., 2014). These latter are raising interest due to their prebiotic potential, as they may (i) decrease the risk of infections and diarrhea, (ii) increase bowel function and metabolism, and (iii) pass through the gastro-intestinal tract and stimulate the growth of resilient beneficial bacteria, mainly the bifidobacteria (Rastall and Gibson, 2014). Apart from their postulated health benefit, prebiotic oligosaccharides may be used in a wide range of applications in clinical, cosmetics, food and feed industries as sweeteners, humectants, possible weight controlling agents and dietary fibers (Patel and Goyal, 2011).

The dextrans produced by *Weissella* spp. have similar structures with mainly (ca. 97%) α-(1-6) linkages and only ca. 3% α-(1-3) linkages (Katina et al., 2009; Bounaix et al., 2010; Maina et al., 2011, 2013; Ahmed et al., 2012; Bejar et al., 2013). This makes dextran-producing strains of *W. cibaria* and *W. confusa* very appealing for a wide range of industrial applications, especially for bakery applications (Di Cagno et al., 2006; Schwab et al., 2008; Katina et al., 2009; Coda et al., 2010, 2014; Galle et al., 2010, 2012; Ruehmkorf et al., 2012; Wolter et al., 2014; Kajala et al., 2015) and for the production of cereal-based, LAB fermented functional beverages (Zannini et al., 2013).

## Isolation, identification, typing, and detection

Pepe et al. (2001) differentially isolated and enumerated *W. paramesenteroides* on Modified Chalmers Agar on which convex colonies of 2 mm with pale-pink colonies containing a small fuchsia center are formed. Zamudio-Maya et al. (2008) used an enrichment in MRS broth followed by plating on MRS agar added with 2,3,5-triphenyltetrazolium chloride (TTC), which allowed the differential isolation of LAB including several *W. confusa* and *W. cibaria* strains from sediments of a coastal marsh. However, apart from these two descriptions, there are no differential selective media available so far for isolation and enumeration of weissellas. Media for presumptive lactobacilli and leuconostocs such as MRS (De Man et al., 1960), which is generally used to cultivate weissellas, LUSM (Benkerroum et al., 1993) and SDB (Kline and Sugihara, 1971) have also been used. Due to the use of vancomycin in the LUSM medium, it may be considered the most selective and useful medium among those mentioned above, although it does not differentiate vancomycin-resistant *Leuconostoc* from weissellas. As for other lactic acid bacteria, the biochemical identification of *Weissella* species, apart from being time-consuming and labor intensive, may be uncertain or lead to misidentification, especially for species with very similar phenotypes. *Weissella* species have previously been distinguished by comparison of cellular fatty acids profiles (Samelis et al., 1998), by total soluble cell protein patterns (Dicks, 1995; Tsakalidou et al., 1997), and furthermore by biochemical-based commercial identification kits such as the RapID™ STR System (Thermo Scientific, Hudson, NH, USA), the API 50 CHL kit (BioMérieux, Lyon, France) (Lee et al., 2012), the Phoenix Automated Microbiology System (Becton DickinsonDiagnostic Systems, Sparks, MD) and the Vitek 2 system (BioMerieux, Marcy l'Etoile, France). But even these advanced tools may fail in discriminating closely related species, due to the variability and instability of phenotypic characters and the subjectivity in the interpretation of results (Graef et al., 2005; Koort et al., 2006; Shin et al., 2007; Kulwichit et al., 2008; Fusco et al., 2011; Lee et al., 2011; Fairfax et al., 2014; Medford et al., 2014). Such is the case, for example, with the two closely related species *W. cibaria* and *W. confusa*, which differ in the capability of the latter to ferment galactose and xylose, while *W. cibaria* produces acid only from L-arabinose (Björkroth et al., 2002; Fusco et al., 2011). To overcome these drawbacks, molecular methods such as 16S rRNA gene sequencing (Kulwichit et al., 2008; Fairfax et al., 2014; Medford et al., 2014), amplified ribosomal DNA restriction analysis (ARDRA) (Jang et al., 2002) and ribotyping (Björkroth et al., 2002) have been used to identify and detect *Weissella* species. Schillinger et al. (2008) designed a *Weissella* and *Leuconostoc* genus specific primer set targeting in the 16S rRNA gene, while Fairfax et al. (2014) used Matrix-assisted laser desorption ionization Time-of-Flight (Maldi-ToF) to identify two *W. confusa* clinical isolates. Walter et al. (2001) designed a primer set allowing the PCR amplification of 16S rRNA gene fragments of the genera *Lactobacillus*, *Pediococcus*, *Leuconostoc*, and *Weissella*, whose separation by denaturing gradient gel electrophoresis resulted in the detection of numerous species belonging to these genera. However, it should be mentioned that, using a gel with a 32.5–40% gradient of urea and formamide increasing in the direction of electrophoresis, a co-migration of the band relevant to *W. confusa* and *Lb. reuteri* was obtained (Walter et al., 2001).

A species-specific PCR, which has been used for the identification and detection of *W. confusa* from foods and clinical specimens, has also been designed (Fusco et al., 2011). Moreover, Snyder et al. (2014) developed a conventional PCR and a quantitative PCR for identification and quantification of *W. ceti* NC36 from pure cultures and tissue samples.

The molecular typing of weissellas was achieved by numerical analysis of *Hind*III and *Eco*RI ribopatterns (Koort et al., 2006), repetitive element-PCR fingerprinting using (GTG)_5_-PCR (Bounaix et al., 2010) and fluorescent-Amplified Fragment Length Polymorphism (fAFLP) (Fusco et al., 2011). Chelo et al. (2010) analyzed genome diversity in the genera *Fructobacillus*, *Leuconostoc*, and *Weissella* by constructing physical and genetic maps, based on pulsed field gel electrophoresis (PFGE) analysis of macro-restriction fragments and hybridization of genetic markers of several strains belonging to these genera. This provided further insights into the evolution and diversification of the species of the genera *Leuconostoc, Oenococcus*, and *Weissella*.

## Description of species of the genus *Weissella*

Currently, the genus *Weissella* consists of 19 species (see below). A detailed description of the currently valid described *Weissella* species is given below:

### *Weissella beninensis* (Padonou et al., 2010)

ben.in.en'sis.N.L.fem.adj. *beninensis*, pertaining to Benin.

*Weissella beninensis* is currently the only known motile species of *Weissella*. Motility was observed by phase contrast microscopy and peritrichous flagella could be visualized by scanning electron microscopy (Padonou et al., 2010). Clustering analysis based on 16S rRNA gene sequences showed *W. beninensis* to cluster with *W. ghanensis* as its nearest neighbor (Padonou et al., 2010), while DNA:DNA hybridization experiments showed that *W. beninensis* was a distinct species, when compared to the nearest neighbor *W. ghanensis*. Cells grow at 15°C but not at 45°C, at a pH range between 3.9 and 8.0 and in medium with 4% NaCl. Ammonia is produced from arginine and gas from glucose catabolism. Both the d and the l lactic acid enantiomers are produced as end products of glucose fermentation. Acid is produced from galactose, lactose, melibiose, raffinose and sucrose, but not from arabinose and xylose. The mol% G+C content is 37.0–37.2%.

### *Weissella ceti* (Vela et al., 2011)

ce.ti. L. gen. n. *ceti*, of a whale.

Bacteria are short rod-shaped or cocci and non-motile. They grow in the presence of 3.0–6.5% NaCl, at pH 3.9 and 37°C, but not at 15 or 42°C (Vela et al., 2011). Both the D and the L lactic acid enantiomers are formed at a ratio of 80:20, respectively. Gas is not produced from glucose metabolism (Vela et al., 2011) which is unusual for a species of this group of organisms as these are all obligately heterofermentative and thus generally should generate gas from glucose fermentation. A 16S rRNA gene sequence analysis showed that *W. ceti* grouped together with *W. halotolerans*, *W. viridescens* and *W. minor* in a well-defined cluster (Vela et al., 2011). Hydrolysis of arginine is variable and strain dependent. Acid is produced from ribose, trehalose and maltose, but not from xylose, galactose, fructose, cellobiose, lactose, sucrose and raffinose. Dextran is not formed from sucrose and both the d and l enantiomers of lactic acid are produced. The mol% G+C content of the DNA is 39.2%. DNA:DNA hybridization was not done to confirm the novel species status of *W. ceti*, despite a high (99.5%) similarity of the 16S rRNA gene sequence to that of other *Weissella* gene sequences in the database (not specified in the publication by Vela et al., 2011).

### *Weissella cibaria* Björkroth et al. (2002, p. 147^VP^)

ci.ba'ri.a.L.adj.*cibaria*, pertaining to food.

*Weissella cibaria* strains originating from Thai fermented foods or from clinical samples were described by Björkroth et al. (2002). These authors noticed that a group of *W. confusa* strains possessed closely related protein fingerprinting patterns and ribotypes, but could nevertheless be distinguished into two distinct groups. These strains were investigated further using, amongst other techniques, 16S rRNA gene analyses and DNA:DNA hybridization and the novel species *W. cibaria*, which is closely related to *W. confusa*, could be distinguished. *W. cibaria* is able to grow at 15 and at 45°C, but not at 4°C (Björkroth et al., 2002). The bacteria tolerate the presence of 6.5% NaCl. *W. cibaria* strains hydrolyse arginine and produce both the d and l lactic acid enantiomer as end product of glucose fermentation. CO_2_ is also generated from glucose metabolism. Acid is produced from arabinose, cellobiose, salicin, sucrose and xylose, but not from galactose, lactose, melibiose, raffinose, ribose and trehalose. Dextran is formed from sucrose. The mol% G+C content of the DNA is 44–45% (Björkroth et al., 2002).

In 2002, Choi et al. described a novel species *W. kimchii* isolated from a traditional vegetable fermentation in Korea. This species was described on the basis of DNA:DNA hybridization, 16S rRNA gene phylogenetic analyses, as well as phenotypic and biochemical testing. The strain was shown to be very similar to *W. confusa*, but differing from this species on the basis of phenotypic characteristics, whole cell protein patterns and DNA:DNA hybridization data (Choi et al., 2002). The species *W. kimchii* was, however, re-classified as *W. cibaria* by Ennahar and Cai (2004), as *W. kimchii* was shown to be a later heterotypic synonym of *W. cibaria* based on 16S rRNA gene sequencing and DNA:DNA hybridization tests. The publication of Choi et al. (2002) in which *W. kimchii* was first described did not compare this species to *W. cibaria* (Björkroth et al., 2002), probably because the authors did not yet have knowledge of the *W. cibaria* species. The latter was also published in 2002, albeit at an earlier time. Therefore, given the earlier publication of the *W. cibaria* description in a work on similar bacteria, Ennahar and Cai (2004) proposed *W. kimchii* to be a later heterotypic synonym of *W. cibaria*.

### *Weissella confusa* Collins et al. (1993, p. 599^AL^)

Synonyms: *Lactobacillus confusus* Sharpe et al. (1972, p. 396); *Lactobacillus coprophilus* subsp. *confusus* Holzapfel and Kandler (1969, 665).

con.fu'sus.L.v. *confundere*; L. past part. *confusus* confused.

These bacteria are heterofermentative and produce both the d and l lactic acid enantiomers when fermenting glucose. Cells are short rods which tend to thicken at one end. The ability to grow at 45°C is strain dependent, with some strains showing good growth at this temperature. Ammonia results from arginine breakdown and acid is produced from cellobiose, galactose, ribose, salicin, sucrose and xylose, but not from arabinose, lactose, melibiose, raffinose and trehalose fermentation. Dextran is formed from sucrose. The mol% G+C content of the DNA is 45–47% (Collins et al., 1993).

### *Weissella diestrammenae* (Oh et al., 2013)

di.es.tram.me'nae. N.L. gen. n. *diestrammenae* of Diestrammena, referring to *Diestrammenacoreana*, a camel cricket from the gut from which the bacteria were isolated.

Cells are coccoid- or rod-shaped and growth occurs from 4 to 37°C, in 0–4% (w/v) NaCl and at pH 5-8 (Oh et al., 2013). Bacteria are heterofermentative and produce gas from glucose, they are able to hydrolyse arginine and produce gas from glucose. The cell wall contains Lys-Ala-Ser and cells produce the d-enantiomer of lactic acid. The mol% G+C content of the DNA is 45% and acid is produced from mannose, acetylglucosamine, xylose and maltose but not from fructose, mannitol, and galactose. Cells are able to hydrolyse esculin and to produce ammonia from arginine (Oh et al., 2013).

### *Weissella fabalis* (Snauwaert et al., 2013)

fa.ba'lis. L. fem. adj. *fabalis* of or belonging to beans.

Bacteria were isolated from fermenting cocoa and 16S rRNA gene sequence analysis showed that this bacterium was most closely related to *W. fabaria* and occurred together with this species, *W. beninensis* and *W. ghanensis* in a well-separated cluster (Snauwaert et al., 2013). Cells are non-motile cocci, which produce gas from glucose in a heterofermentative metabolism. This bacteria produces the d lactic acid enantiomer, grows at 15–37°C and in the presence of 5–6% NaCl, but not in the presence of 7–8% NaCl (Snauwaert et al., 2013). Acid is formed from fructose, cellobiose, trehalose and gentiobiose, but not from arabinose, ribose, xylose, galactose, lactose, melibiose, sucrose and raffinose. Arginine is hydrolyzed. The mol% G+C content is 37% (Snauwaert et al., 2013).

### *Weissella fabaria* (De Bruyne et al., 2010)

fa.ba'ri.a. L. fem. adj. *fabaria*, of or belonging to beans.

Bacteria were also isolated from fermenting cocoa and are heterofermentative producing CO_2_ from glucose metabolism. They furthermore produce both the d and l lactate enantiomer in an approximate 90:10 ratio, respectively (De Bruyne et al., 2010). Cells are non-motile, coccoid with growth occurring at 15–37°C and at pH 5.0–9.0. No growth occurred in the presence of 5% NaCl. *W. fabaria* hydrolyses arginine and acid is produced from fructose, mannose, cellobiose, trehalose and gentiobiose, but not from arabinose, ribose, raffinose, sucrose, xylose, galactose, lactose, and melibiose. According to a 16S rRNA gene sequence analysis, *W. fabaria* was shown to be closely related to *W. ghanensis* and occurred together with this species, as well as with *W. fabalis* and *W. beninensis* in a well-delineated cluster (De Bruyne et al., 2010; Snauwaert et al., 2013; Björkroth et al., 2014). The mol% G+C content of the DNA is 38.2% (De Bruyne et al., 2010).

### *Weissella ghanensis* (De Bruyne et al., 2008)

gha.nen'sis.N.L.fem.adj. *ghanensis*, pertaining to Ghana.

*Weissella ghanensis* was also isolated from fermenting cocoa. These bacteria are small rods, appearing singly or in chains and are non-motile. *W. ghanensis* grows at 15–37°C, but similar to *W. fabaria*, it does not grow in the presence of 5% NaCl (De Bruyne et al., 2008). The strain produces gas (CO_2_) from glucose fermentation, with both the d and l lactic acid enantiomers being produced at a ratio of approx. 90:10 or 95:5, respectively, depending on the strain (De Bruyne et al., 2008).*W. ghanensis* hydrolyses esculin and produces ammonia from arginine. Acid is produced from cellobiose, fructose, maltose, salicin and trehalose, with no acid being produced from arabinose, galactose, melibiose, raffinose, ribose and xylose. The mol% G+C content of the DNA is 40%.

### *Weissella halotolerans* Collins et al. (1993, p. 599^VP^)

Synonym: *Lactobacillus halotolerans* Kandler et al. (1983). (p. 672). Effective publication: Kandler et al. (1983); Kandler et al. (p. 283).

ha.lo.to'le.rans. Gr. N. *hals*, *halos*, salt; L. part. adj. *tolerans*, tolerating, enduring: N.L. part. adj. *halotolerans*, salt tolerating.

*Weissella halotolerans* was originally described as “*Lactobacillus viridescens* subsp. *halotolerans*” by Reuter (1970), but this name was not on the *Approved List of Bacterial Names* of Skerman et al. (1989). “*Lactobacillus halotolerans*” was subsequently described by Kandler et al. (1983) as irregular short, even coccoid rods with rounded ends and with a tendency to form coiling chains and lumping together. Growth of these bacteria occurred between 10 and 40°C, with good growth occurred only from 12°C. Very weak growth could be demonstrated at 14% NaCl. “*L. halotolerans”* was shown to ferment fructose, glucose, gluconate, maltose, mannose, ribose and trehalose, but not cellobiose, galactose, lactose, mannitol, melizitose, melibiose, raffinose, rhamnose, sorbitol, sucrose, xylose and esculin. The cell wall murein type was Lys-Ala-Ser and the mol% G+C content of the DNA was 45% (Kandler et al., 1983). Some 10 years later, Collins et al. (1993) finally reclassified “*Lactobacillus halotolerans*” as *Weissella halotolerans*.

### *Weissella hellenica* Collins et al. (1993, p. 601^VP^)

hel.en'i.ca.Gr.adj.*Hellenikos*, Greek; N.L. fem. adj. *hellenica*, Greece, from where this bacterium was first isolated.

Bacteria are non-motile and of spherical, but sometimes lenticular shape, usually occurring in pairs or short chains, with a tendency to form clusters (Collins et al., 1993). Cells grow at 10°C, show delayed growth at 4°C and do not grow at 37°C. The bacteria are heterofermentative but gas production is poor. The cells produce predominantly (>98%) d-lactate, they do not hydrolyse arginine. Growth occurs at 8% but not at 10% NaCl, or in medium at pH 4.8–5.0. They are Vogues-Proskauer negative. Acid is produced from glucose, fructose, mannose, maltose, trehalose but not from ribose, xylose, rhamnose, mannitol, cellobiose, lactose, melizitose and raffinose. The cell wall peptidoglycan belongs to the Lys-Ala-Ser(Ala)- type and the G+C content of the DNA was reported to range from 39.4 to 40 mol% (Collins et al., 1993).

### *Weissella kandleri* Collins et al. (1993, p. 599^VP^)

Synonym: *Lactobacillus kandleri* Holzapfel and van Wyk (1983) (p. 439). Effective publication: Holzapfel and van Wyk (1982); Holzapfel and van Wyk (p. 501).

kand'le.ri. M.L. gen. n. *kandleri*, of Kandler; named after O. Kandler, a German microbiologist.

Cells are of irregular rod shape occurring singly or in pairs. The interpeptide bridge is Lys-Ala-Gly-Ala_2_. Both the d- and the l-enantiomers of lactate are produced. Dextran is generated from sucrose, ammonia is produced from arginine. No growth occurs at 45°C. The G+C content of the DNA is 39 mol%. Acid is produced from galactose, ribose but not from arabinose, cellobiose, maltose, melibiose, raffinose, sucrose, trehalose and xylose (Holzapfel and van Wyk, 1983).

### *Weissella koreensis* Lee et al. (2002). (p. 1260^VP^)

ko.re.en'sis.N.L.*koreensis* of Korea.

*W. koreensis* was isolated from Korean kimchi and is most closely related to *W. kandleri* on basis of a 16S rRNA gene nucleotide analysis. *W. koreensis* bacteria are not motile and the cells are irregular, short and rod-shaped or coccoid. These bacteria were able to grow at 10 and 37°C but not at 42°C. Cells also grew in a pH range of pH 4.0–8.0, but not in the presence of 8 or 10% NaCl. Arginin was hydrolysed and acid was produced from arabinose, ribose and xylose, but not from cellobiose, galactose, maltose, melibiose, raffinose, sucrose or trehalose. The bacteria form exclusively the d lactic acid enantiomer. The cell wall was shown to contain Lys-Ala-Ser and the mol% G+C content of the DNA was 37 mol% (Lee et al., 2002).

### *Weissella minor* Collins et al. (1993, p. 599^VP^)

Synonyms: *Lactobacillus minor* Kandler et al. (1983). (p. 672). Effective publication: Kandler et al. (1983). (p. 284) (*Lactobacillus corynoides* subsp. *minor* Abo-Elnaga and Kandler (1965) (p. 128); *Lactobacillus viridescens* subsp. *minor* Kandler and Abo-Elnaga (1966) (p. 754).

mi'nor. L. comp. adj. *minor* smaller.

*W. minor* was previously classified as a subspecies of *Lactobacillus viridescens*, i.e., *L. viridescens* subsp. *minor* (Abo-Elnaga and Kandler, 1965), similar to the case of *W. halotolerans* which was previously classified as *Lactobacillus viridescens* subsp. *halotolerans* (Reuter, 1970; Kandler et al., 1983). These bacteria were re-classified to “*Lactobacillus minor”* by Kandler et al. (1983) who described these bacteria as occurring as irregular, short rods with rounded to tapered ends, often bent and with unilateral swelling. Bacteria were non-motile and were able to grow between 10 and 40°C. Good growth could be shown to occur up to 8% NaCl, and very weak growth at 10% NaCl. Nitrate was not reduced to nitrite, the cell wall contained murein of the Lys-Ser-Ala_2_ type and the mol% G+C content of the DNA was 44 mol%. The bacteria were able to produce acid from cellobiose, fructose, glucose, maltose, mannose, melizitose, ribose, sucrose, trehalose and esculin, but not from arabinose, galactose, lactose, mannitol, melibiose, raffinose, rhamnose, sorbitol, and xylose (Kandler et al., 1983).

### *Weissella oryzae* (Tohno et al., 2013)

o.ry'za.e.L.gen.n. *oryzae* of rice, from which the type strain was isolated.

These bacteria from fermented rice grains were irregular, short rod-shaped or cocci and occurred singly or in pairs or short chains. Cells grew at 10–42°C, but not at 4 or 50°C. Growth also occurred at pH 3.9–9.0 and with 4.0–6.5% NaCl, but not with 8% NaCl (Tohno et al., 2013). The cell wall peptidoglycan contains glutamic acid, lysine, serine and alanine and acid was produced from arabinose, ribose, xylose, galactose (delayed reaction), glucose, fructose, mannose, maltose, melibiose, trehalose and gluconate. Acid was not generated from arabinose, xylose, sorbose, rhamnose, dulcitol, inositol, mannitol, esculin, cellobiose, lactose, sucrose, melizitose, and raffinose. Esculin was not hydrolyzed, arginine hydrolysis was positive. Only the d lactic acid enantiomer was formed from carbohydrate fermentation. The mol% G+C content of the DNA was 40.6 mol%.

### *Weissella paramesenteroides* (Garvie, 1967; Collins et al., 1993) comb. nov.

Gr. prep.*para*, beside; N.L. fem. adj. *mesenteroides*, a specific epithet; N.L. fem. adj. *paramesenteroides*, beside *Leuconostoc mesenteroides*.

*Weissella paramesenteroides* was proposed as a new species of the genus *Leuconostoc* by Garvie in 1967 based on differences to the closely related *Leuconostoc mesenteroides* regarding amino acid and vitamin requirements, and the failure to hydrolyze esculin and salicin. Morphologically, these bacteria are very similar to *L. mesenteroides* and do not form dextran from sucrose (Garvie, 1967) or ammonia from arginine. They are more tolerant toward NaCl than *L. mesenteroides* and can grow in media with an initial pH below 5. The optimum growth temperature is 18–24°C, but many strains also grow at 30°C. The bacteria produce acid from galactose, maltose, melibiose, sucrose and trehalose. The peptidoglycan type is Lys-Ala_2_or Lys-Ser-Ala and the G+C content of the DNA is 37–38 mol%. Based on 16S rRNA gene sequencing analysis, the leuconostocs were shown to comprise three distinct lineages of which the “*L. paramesenteroides”* group (including the species formerly known as “*L. confusus,” “L. minor,” “L. kandleri,” “L. halotolerans,”* and “*L. viridescens”*) were re-assigned to the genus *Weissella* by Collins et al. (1993). The re-classification of this new group included the new species *W. paramesenteroides* (Collins et al., 1993).

### *Weissella soli* (Magnusson et al., 2002)

so'li.L.n. solum soil; L.gen.n.*soli* of the soil.

*W. soli* was isolated from garden soil in Uppsala, Sweden. Bacteria are non-motile rods that are thickened at one end and occurred singly or in pairs. The lactic acid enantiomer produced was mainly d-lacticacid. Growth occurred between 4 and 40°C, but not at 42°C. Acid was produced from ribose, D-xylose, glucose, mannose, maltose, melibiose, sucrose, trehalose and raffinose, but not from l-xylose, galactose, fructose, rhamnose, mannitol, cellobiose, lactose, and melizitose. Esculin was shown to be hydrolyzed and arginine was cleaved. The G+C content of the DNA was 43%mol (Magnusson et al., 2002).

### *Weissella thailandensis* (Tanasupawat et al., 2000)

thai.lan'den.sis.M.L.fem. adj. *thailandensis* pertaining to Thailand, where the strains were first isolated.

*W. thailandensis* was isolated from fermented fish (pla-ra) in Thailand and cells were non-motile and coccus-shaped. These bacteria did not reduce nitrate and did not hydrolyze arginine, esculin, gelatin or starch. The bacteria were able to grow in 10% NaCl, at temperatures of 25–37°C but not at 42°C. Growth also occurred at pH 8.0, while no growth could be observed at pH 4.5 or pH 8.5. Acid was produced from ribose, arabinose, fructose, galactose, mannose, maltose, melibiose, raffinose, and rhamnose, but not from cellobiose, mannitol, melizitose, sorbitol or xylose. The cell wall contains Lys-Ala_2_ and the mol% G+C content in the DNA was found to range from 38 to 41.2 (Tanasupawat et al., 2000).

### *Weissella uvarum* Nisiotou et al., 2014

u.va'rum.L.fem. gen. pl. n. *uvarum* of grapes, where the type strain was isolated.

This bacterium was isolated from grapes from Nemea in Grecce. The cells were non-motile short rods, or showed coccoid morphology. The cells were able to grow at 15 and 42°C, but not at 4 or 45°C. Cells were not capable of growth at pH 3.9 or in the presence of 6.5% NaCl, but could grow at pH 8.0 and in the presence of 4% NaCl. Ammonia was produced from arginine and acid was produced from ribose, glucose, fructose, mannose, trehalose, melizitose, but not from glycerol, arabinose, xylose, galactose, rhamnose, mannitol, sorbitol, esculin, cellobiose, maltose, lactose, melibiose, sucrose, and raffinose (Nisiotou et al., 2014). The mol% G+C content of the DNA and amino acid composition of the cell wall were not yet determined.

### *Weissella viridescens* (Niven and Evans., 1957; Collins et al., 1993) comb. nov.

vi.ri.des'cens. M. L. pres. part.*viridescens*, growing green, greening.

The bacteria previously known as *Lactobacillus viridescens* were re-classified as *Weissella viridescens* by Collins et al. (1993). Cells are small rods, which occur either singly or in pairs, and the ends of the rods appear slightly tapered. Nitrate is not reduced and sodium hippurate, esculin, arginine and starch are not hydrolyzed. Growth occurs in the presence of 6.5% NaCl and at the low temperature of 5°C, but not at 45°C. These bacteria ferment glucose, mannose, fructose and maltose, but no acid is produced from xylose, arabinose, galactose, lactose, raffinose or sorbitol (Niven and Evans, 1957). The interpeptide bridge of the peptidoglycan is composed of Lys-Ala-Ser. The mol% G+C of the DNA is 41–44 (Kandler and Weiss, 1986). Some strains produce large amounts of dextran from sucrose fermentation, a trait that may be lost rapidly in stock cultures (Niven and Evans, 1957).

## Conclusions

The genus *Weissella* is a well-delineated genus within the family *Leuconostocaceae* and contains 19 validly described species. *Weissella* species are generally difficult to distinguish from other heterofermentative cocci such as leuconostocs, or rod-shaped bacteria such as certain *Lactobacillus* strains on the basis of phenotypic or biochemical properties alone. An accurate species identification thus is generally only possible using molecular biological methods, such as sequencing of 16S rRNA or other house-keeping genes, DNA:DNA hybridization and by typing methods such as rep-PCR or fAFLP. Weissella occur in a great variety of habitats, including the skin, milk and feces of animals, the saliva, breast milk, feces and vagina of humans, on plants and vegetables, as well as in a variety of fermented foods such as European sourdoughs and Asian and African traditional fermented foods. Strains of some *Weissella* species, e.g., of *W. viridescens*, *W. cibaria*, and *W. confusa*, are known as opportunistic human pathogens causing infections such as bacteremia and endocarditis. *Weissella ceti* has also recently been shown to be the causative agent of “weissellosis” in farmed rainbow trout (*Oncorhynchus mykiss*) causing septicemia with a high mortality rate. On the other hand, there is some interest for biotechnological application of these bacteria, as specific strains have been investigated for use as probiotics, also for combatting periodontal disease. As *Weissella* strains feature quite prominently in some African fermented foods, or in European sourdough fermentations, the exploitation of specific strains as starter cultures may be considered. One technological interesting feature is also the production of copious amounts of dextrans and novel extracellular polysaccharides with potential prebiotic activity. This may be important for use in bakery products, for development, e.g., of cereal-based, LAB fermented functional beverages, or potentially for improving the viscosity and mouth-feel of fermented foods such as, e.g., fermented milk products. However, food producers should always be cognizant of the potential of *Weissella* strains for human opportunistic infection, and safety testing of any strain before use in biotechnological application is mandatory.

### Conflict of interest statement

The authors declare that the research was conducted in the absence of any commercial or financial relationships that could be construed as a potential conflict of interest.
